# Long-lasting postoperative analgesia with local anesthetic-loaded hydrogels prevent tumor recurrence via enhancing CD8^+^T cell infiltration

**DOI:** 10.1186/s12951-023-01803-8

**Published:** 2023-02-10

**Authors:** Mingxu Zhao, Shasha Zhu, Ding Zhang, Chang Zhou, Zhilai Yang, Chunhui Wang, Xuesheng Liu, Jiqian Zhang

**Affiliations:** 1 Department of Anesthesiology, Key Laboratory of Anesthesia and Perioperative Medicine of Anhui Higher Education Institutes, The First Affiliated Hospital of Anhui Medical University, Anhui Medical University, Hefei, 20032 China; 2 Department of Anesthesiology, The Fourth Affiliated Hospital of Anhui Medical University, Hefei, 20032 China; 3 Reproductive Medicine Center, Department of Obstetrics and Gynecology, The First Affiliated Hospital of Anhui Medical University, Hefei, 20032 China; 4 Department of Anesthesiology, The First Affiliated Hospital of Anhui University of Chinese Medicine, Hefei, 20032 China

**Keywords:** Postoperative pain, Local anesthetic, Tumor recurrence, MHC-I, Hydrogel

## Abstract

Postoperative pain (POP) can promote tumor recurrence and reduce the cancer patient's quality of life. However, POP management has always been separated from tumor treatment in clinical practice, and traditional postoperative analgesia using opioids is still unsatisfactory for patients, which is not conducive to tumor treatment. Here, ropivacaine, a popular amide-type LA, was introduced into a Pluronic F127 hydrogel. Postoperative analgesia with ropivacaine-loaded hydrogels reduced the incidence of high-dose ropivacaine-induced convulsions and prolonged pain relief for more than 16 h. More interestingly, ropivacaine-loaded hydrogel was found to upregulate major histocompatibility complex class I (MHC-I) in tumor cells by impairing autophagy. Therefore, a hydrogel co-dopped with ropivacaine and TLR7 agonist imiquimod (PFRM) was rationally synthesized. After postoperative analgesia with PFRM, imiquimod primes tumor-specific CD8^+^T cells through promoting DCs maturation, and ropivacaine facilitates tumor cells recognition by primed CD8^+^T cells through upregulating MHC-I. Consequently, postoperative analgesia with PFRM maximumly increases CD8^+^T cells infiltration into residual tumor tissue and prevents tumor recurrence. Overall, this study for the first time provides an LA-based approach for simultaneous long-lasting postoperative analgesia and prevention of tumor recurrence.

**Supplementary Information:**

The online version contains supplementary material available at 10.1186/s12951-023-01803-8.

## Introduction

Postoperative pain (POP) results from surgical injuries to the skin, fascia, muscle, and the small nerves innervating these tissues and is a common form of acute clinical pain [1]; it has been reported that more than 80% of patients experience acute pain after surgery, and up to 71% of patients experience moderate to extreme pain despite treatment according to acute pain management protocols [2]. POP significantly affects the patient recovery, leading to complications, such as impaired wound healing, insomnia, and anxiety, or to the development of chronic persistent POP, which occurs in 10–50% of patients suffering from acute POP [3]. Notably, close attention should be paid to patients who have undergone cancer surgery in terms of POP management, as extensive studies have reported a correlation between POP and tumor recurrence and metastasis [4]. Currently, opioids are the most popular drugs for POP management; however, they have many dose-limiting side effects such as respiratory depression, nausea, vomiting, pruritus, bowel dysfunction, abuse, and fatal overdose due to misuse [5]. Therefore, analgesic management based on local anesthetics (LAs) such as epidural analgesia [6, 7], peripheral nerve blocks [8, 9], and wound infiltration [10, 11] has attracted increased attention because of their effectiveness, low-cost, and relatively few side effects. However, the short duration of analgesia they provide and their toxicity at high concentrations limit the clinical application of LAs [8, 12, 13]. Engineered LAs can circumvent these disadvantages and have promising application prospects. In clinical practice, Exparel liposomes, XaraColl collagen sponges, POSIMIR solution, and Zynrelef extended-release solution have been approved by the FDA for POP management. However, they have many limitations, such as limited indications, controversial clinical effects, complex preparation procedures, and high cost [14]. Hence, in preclinical studies, researchers have developed various engineered LAs to improve POP management. For example, Ji et al. designed a self-assembled supramolecular system to deliver TTX to significantly extend a nerve block for up to 16 h and significantly reduce TTX toxicity [15]. Zhang et al. found that gel-immobilized bupivacaine-loaded microspheres induced sensory blockades lasting for 24.25 ± 1.28 h [16]. Masaru et al. reported that a single administration of a slow-release lidocaine sheet with polylactic-coglycolic acid relieved incision pain for one week in rats [17]. Carolina et al. found that 0.75% ropivacaine large multivesicular vesicles achieved prolonged analgesic effects in rats that underwent a hind paw incision [18]. Although engineered LAs have performed well in POP management, their performance in pain management after tumor resection has not been assessed in preclinical studies.

In terms of POP, postoperative recurrence linked with higher mortality rates in patients with cancer [19]. Surgery is currently the mainstream clinical strategy for the treatment of most solid tumors [20], but despite advances in surgical techniques, residual tumor cells can remain in the surgical margins or circulation after surgery, which increases the risk of cancer recurrence and metastasis [21]. Additionally, surgery often induces a stress response characterized by metabolic changes, local inflammation, and pain, thus causing an elevation in circulating glucocorticoids and compromising anti-tumor immune responses, which can promote cancer recurrence [4]. Therefore, effective POP management can alleviate the stress response and provide relief for patients. However, studies on painless cancer treatment using LAs are rare. Growing evidence has revealed that LAs can directly or indirectly kill tumor cells and prevent postoperative recurrence, potentially due to direct cytotoxic effects or indirect immune-mediated effects [22, 23]. However, the mechanism of direct modulation of the immune system by LAs is unclear, and research on immunotherapy for cancer using LAs is pending. Dysfunctional antigen presentation due to mutations or loss of heterozygosity of major histocompatibility complex class I (MHC-I) is a common mechanism of tumor cell immune evasion [24]. Interestingly, Keisuke et al. found that the inhibition of autophagy increases MHC-I in tumor cells and results in improved antigen presentation, enhanced anti-tumor T-cell responses, and reduced tumor growth in mice [25]. In addition, our previous study revealed that ropivacaine, one of the most commonly used LAs in clinical practice, inhibited autophagy by impairing lysosomal degradation [26]. Therefore, we hypothesize that ropivacaine upregulates MHC-I in tumor cells and can therefore be used in immunotherapy.

**Fig. 1:**
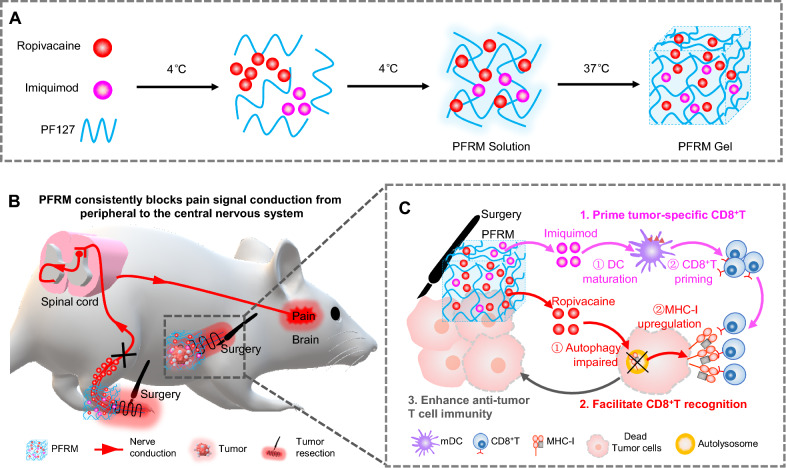
A schematic illustration showing that LA-loaded hydrogels relieve post-tumor resection pain and promote the killing of residual tumor cells by CD8^+^ T cells. **A** Preparation of PFRM hydrogel. **B** PFRM sustainedly blocks the conduction of pain signals from the peripheral to the central nervous system. **C** Mechanism of PFRM preventing tumor recurrence. First, imiquimod promotes the DCs maturation and primes tumor specific CD8^+^ T cells; second, ropivacaine upregulates MHC-I levels in tumor cells and facilitates their recognition by CD8^+^ T cells; consequently, postoperative analgesia with PFRM prevents tumor recurrence through potentiating CD8^+^ T cells immunity

Pluronic F127 (PF127) is an FDA-approved thermoresponsive biocompatible polymer [27]. We prepared PF127 hydrogels doped with ropivacaine (PFR) and tested their analgesic effect after tumor resection surgery. To simulate clinical reality, tumor-bearing mice were sequentially injected with chemotherapy drugs to control tumor growth, and we resected the tumors under general anesthesia. We found that postoperative analgesia with PFR did not cause convulsions, whereas 16.67% of the free ropivacaine-treated mice experienced convulsions. In addition, PFR administration significantly prolonged POP relief for more than 16 h and decreased the number of p-p38 positive neurons in the spinal dorsal horn of mice, suggesting effective POP relief. Furthermore, PFR impaired autophagy and increased MHC-I levels. Upregulation of MHC-I is known to promote the recognition of tumor cells by cytotoxic T lymphocytes (CD8^+^ T cells) [25], revealing the immunotherapeutic potential of PFR. Imiquimod is an FDA-approved TLR7 agonist that reportedly increases the population of CD8^+^ T cells by promoting dendritic cell (DC) maturation [28]. Therefore, a ropivacaine- and imiquimod-co-loaded PF127 hydrogel (PFRM) was prepared, and its effect in preventing tumor recurrence was tested. Indeed, postoperative analgesia with PFRM synergistically primed tumor specific CD8^+^ T cells through promoting DCs maturation, and facilitated CD8^+^ T cells recognition on residual tumor cells through upregulating MHC-I, exhibiting an excellent anti-tumor recurrence effect (Fig. 1). This study for the first time provides an LA-based approach for simultaneous long-lasting postoperative analgesia and prevention of tumor recurrence.

## Materials and methods

### Materials and antibodies

Pluronic F127 (P2443) was purchased from Sigma-Aldrich and ropivacaine (R413090) was obtained from Mackline, while Imiquimod (HY-B0180A) and doxorubicin (Dox, HY-15142) were purchased from MCE. Butorphanol (H20020454) was purchased from Hengrui. Anti-mouse CD45-PE-cy7 (103114), anti-mouse CD11c-APC-cy7 (117323), anti-mouse CD3-APC-cy7 (100221), anti-mouse CD8a-APC (100711), anti-mouse CD16/32-TruStain FcX (101320), anti-mouse CD86-APC (105011), anti-mouse CD11b-PE (101207), anti-mouse Gr1-APC (108411), anti-mouse CD4-PE (100408) and anti-mouse Foxp3-AF488(126406) antibodies were purchased from BioLegend. Anti-mouse CD80-FITC (FW0149), anti-mouse MHC-I-APC (FW2919), and anti-Rb Phospho-p38 (Thr180/Tyr182) (DL3485) were purchased from Elabscience. Anti-LC3 (ab192890) and anti-P62 (ab109012) antibodies and Alexa Fluor 568 and Alexa Fluor 488 secondary antibodies were purchased from Abcam. BD™ Cytometric Bead Array (CBA) Mouse TNF Flex Set (558299), Mouse IFN-γ Enhanced Sensitivity Flex Set (562233) and Mouse Enhanced Sensitivity Master Buffer Kit (562246) were purchased from BD Bioscience.

### Preparation and characterization of PF127 hydrogels

PF127 hydrogels were prepared using the “cold method” as described in a previous report [29]. Briefly, 0.25 g PF127 was mixed with 1 mL of cold water and stirred at 4 °C until completely dissolved. Then, 10 mg of ropivacaine or 0.3 mg of imiquimod was added to 1 mL of PF127 hydrogel and stirred at 4 °C to obtain the desired hydrogel. The morphology of lyophilized hydrogels was observed using a Carl Zeiss field-emission scanning electron microscope (SEM).

### Rheological analysis of hydrogels

Rheological experiments were performed for different aqueous solutions containing ropivacaine or other drugs on Anton Paar (MCR 302) using a parallel plate (plate diameter, 40 mm; gap, 0.5 mm). The storage modulus (G′) and loss modulus (G″) of different combinations were quantified under different conditions. For the temperature-dependent experiments, the heating rate was set at 2.0 °C/min. The sol–gel transition temperature was determined as the intersection point of G′ and G″. On the other hand, frequency-dependent rheological measurements were conducted at 37 °C, with a strain rate of 0.1%. Shear-dependent changes in viscosity was carried out at 37 °C.

### In vitro* release study*

The hydrogel was placed into a glass tube was placed at 37 °C to form a gel. Deionized water (4 mL, 37 °C) was carefully layered on top of the gel, and the tubes were placed in an orbital shaker at 37 °C at 70 rpm. At the indicated times (3, 4, 6, 8, 24, and 48 h), 1 mL of the solution was collected and replaced with an equal volume of deionized water [29]. Finally, concentrations of ropivacaine were determined by testing the absorbance at 268 nm using a micro-UV–Vis spectrophotometer (LIFEREAL).

### Cell culture

First, 4T1 cells were cultured at 37 °C with 5% CO_2_ in Dulbecco’s modified Eagle’s medium (DMEM) containing 10% fetal bovine serum (FBS), 100 U/mL penicillin, and 100 µg/mL streptomycin. To obtain LC3-GFP-infected cells, 4T1 cells were seeded in a glass-bottomed dish and cultured in DMEM containing 5 μL Ad-GFP-LC3B (C3006, Beyotime). The virus-containing medium was replaced with fresh medium within 24 h. Finally, cells were cultured for another 24 h. After PF or PFR treatment for 8 h, cells were stained with 75 nM LysoTracker Red for 15 min to label the lysosomes and then washed twice with PBS. Cells were then observed under a confocal microscope (LSM800, Zeiss). Lysosome size was analyzed using ImageJ software using the “Analyze Particle-Count/Size” tool with default settings. The 4T1 cells were provided by Prof. Longping Wen of the South China University of Technology.

### Western blotting

Cells were lysed using sample buffer and boiled for 10 min. Protein samples were separated using electrophoresis on 13.5% sodium dodecyl sulfate sulfate–polyacrylamide gels and transferred to polyvinylidene difluoride membranes. The membranes were incubated with the primary antibody overnight at 4 °C and with secondary antibody at 37 °C for 1 h. Membranes were incubated with ECL kit reagents and then detected using a chemiluminescence instrument (Amersham Imager 600, GE Healthcare).

### Immunofluorescence

The mice were anesthetized, followed by intracardiac perfusion with 20 mL of PBS and 4% paraformaldehyde, and L3-5 spinal cord segments were obtained. Then, tissues were fixed overnight in 4% paraformaldehyde and treated with 30% sucrose for 24 h. Following this, the tissue was embedded with Tissue-Tek O.C.T. compound and cut into 10-μm sections. The sections were observed using the confocal microscopy.

Cells were fixed with 4% paraformaldehyde for 10 min, permeabilized with 0.1% Triton X-100 for 10 min, and then blocked with 1% FBS for 1 h. Cells were incubated with primary antibody overnight at 4 °C and incubated with the fluorescent secondary antibody for 1 h at 37 °C. After washing and sealing, the sections were observed under a confocal microscope.

### Animals

Six to eight-weeks-old female BALB/c mice weighing 18–25 g were purchased from the Shanghai Laboratory Animals Center. All animals were housed in temperature-, humidity-, and light-controlled rooms and provided with adequate water, and free access to food. Experimental procedures were carried out in accordance with the Ethical Regulations for the Care and Use of Laboratory Animals of Anhui Medical University and were approved by the school animal committee.

### Behavioral assessment

Paw withdrawal latency (PWL) was measured by placing individual mice on the glass surface of a thermal testing apparatus (Model 336; IITC/Life Scientific Instruments, Woodland Hills, CA, USA). Before each measurement, the mice were placed in the apparatus for 30 min to acclimatize to the environment. A removable thermal stimulator concentrated heat on the left hind paw wound of the mouse through a hole in the light box through the glass plate. When the animal lifted or licked its hind paw, the light beam was turned off. The time interval between the beginning and end of thermal stimulation was defined as the PWL. Each experiment was repeated three times with a 5 min interval between each replicate. A cutoff time of 20 s was set to avoid injury [30].

Following this, 4T1 cells (2 × 10^5^, 20 μL) were injected into the plantar region of the left hind paw of each mouse to construct the tumor-bearing mice model [31]. DOX (2 mg/kg) was injected into the tumor for three days before surgery. Seven days after the tumor-cell inoculation, the mice underwent tumor excision surgery. All mice were subcutaneously injected with 20 μL of butorphanol (1 mg/mL) 30 min before surgery. PBS, free ropivacaine, or PFR (30 μL) were injected to the incision site for postoperative analgesia. PWL was measured as the baseline value before surgery and then measured again at indicated times.

### Anti-tumor recurrence assessment

To observe the anti-tumor recurrence effect of PFRM, 8 × 10^5^ 4T1 cells were inoculated into the right breast pad of the BALB/c mice. DOX was intratumorally injected for three days before surgery, and then the tumors were removed [32, 33] when the tumor volume reached approximately 150 mm^3^. The mice underwent tumor resection under isoflurane general anesthesia, and they were subcutaneously injected with 20 μL of butorphanol for intraoperative analgesia. After surgery, mice were randomly divided into four groups and injected with 150 μL of PBS, PFR, PFM, or PFRM in situ every other day for a total of three times. In addition, the body weight of mice and the recurrence of tumors in the surgical incision were measured every other day, and the tumor volume was calculated using the following formula: volume (mm^3^) = 0.5 × width^2^ (mm^2^) × length (mm) [34].

### Flow cytometry analysis

To detect MHC-I, 4T1 cells were seeded into 24-well plates and incubated with PF or PFR (40 μL added to 500 μL culture medium) for 8 h. Cells were harvested and stained with APC anti-MHC-I antibody. The MFI of MHC-I was determined using a flow cytometer (BD Verse).

To assess DC maturation, postoperative mice were injected with the indicated hydrogels for analgesia. Three days after the final dose of analgesia, the draining lymph nodes (LNs) were harvested for DC maturation analysis by counting the percentage of CD11c^+^ CD80^+^ and CD11c^+^ CD86^+^.

To assess CD8^+^ T cell, MDSCs and Tregs infiltration in tumors, tumor-bearing mice were treated as described above. At the end point, tumors were harvested, and the percentage of CD45^+^CD3^+^CD8^+^, CD11b^+^Gr1^+^ and CD4^+^Foxp3^+^ cells were calculated using flow cytometry. The levels of TNF-α and IFN-γ were measured using cytometric bead assay (CBA) according to the manufacturer’s protocols.

### Statistical analysis

Data are presented as the mean ± standard deviation (SD). Comparisons between the two data sets were analyzed using a two-tailed Student’s t-test. Comparisons between three or more groups of data were analyzed using one-way or two-way repeated-measure analysis of variance with Tukey’s post hoc test. Differences of P < 0.05, P < 0.01, and P < 0.001 were considered statistically significant and are labeled with *, **, and ***, respectively.

## Results and discussion

### Ropivacaine-loaded hydrogel relieves pain post-tumor resection

**Fig. 2:**
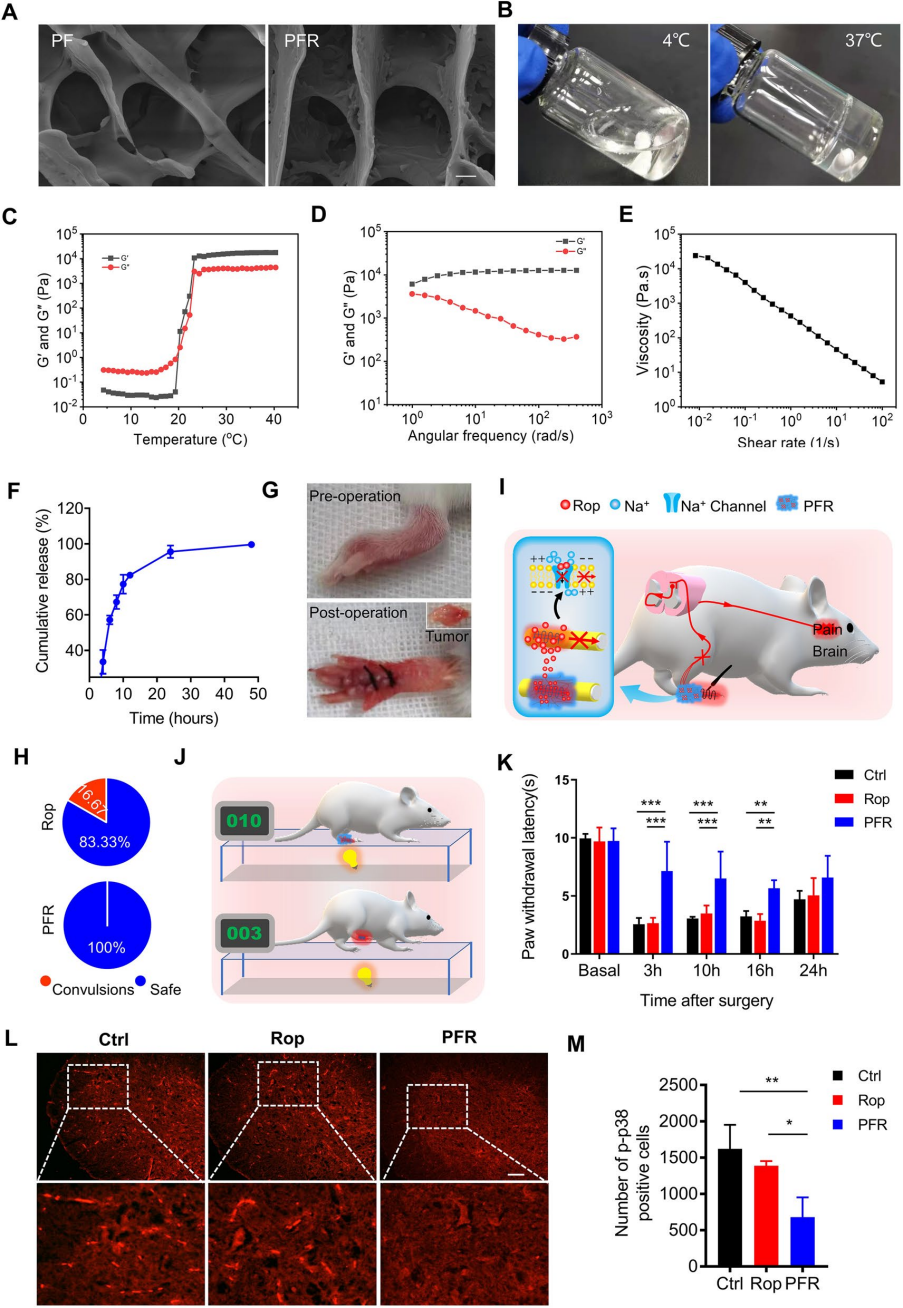
PFR prolongs the duration of postoperative analgesia. **A** SEM images of PF and PFR (scale bar represents 2 μm). **B** Photos of PFR at 4 °C and 37 °C. **C** Temperature-dependent rheology of PFR aqueous dispersion. **D** Frequency-dependent rheology of PFR hydrogel at 37 °C. **E** The shear-thinning behavior of PFR hydrogel indicated by steady-shear rheology. **F**Cumulative release profile of PFR. **G** Photos of plantar tumor before and after surgery. **H** Statistical results of the occurrence of convulsions in post operative mice administrated with PFR or free ropivacaine. **I** Schematic diagram of postoperative pain relief by PFR. PFR slowly releases ropivacaine, which blocks sodium channels and inhibits the conduction of surgery-triggered painful stimuli via afferent nerves. **J** Schematic diagram of thermal hyperalgesia assessment. The time to withstand thermal stimulation was recorded. **K** Paw withdrawal latency was measured at 0, 3, 10, 16, and 24 h after tumor resection with indicated analgesia (n = 5). **L**–**M** Immunofluorescence staining and statistical results of p-p38 in the spinal dorsal horn at three days after plantar tumor resection (scale bar represents 100 μm, n = 3). *Rop* ropivacaine, *PFR* PF hydrogel mixed with ropivacaine

In clinical practice and preclinical studies, LAs have been used to manage postoperative pain [35–37]. However, the short duration of analgesia they provide and their high toxicity at high concentrations severely limits the clinical application of LAs [8, 12, 13]. To overcome these disadvantages, we mixed ropivacaine, a popular amide-type LA, with a PF127 hydrogel to form a PFR hydrogel (ropivacaine 1% and PF127 25%, w/v). A porous structure of PF127 and PFR was observed using SEM (Fig. 2A). Furthermore, the PFR solution formed a free-flowing transparent liquid at 4 °C and showed a clear hydrogel formation at 37 °C (Fig. 2B), which is suitable for in vivo implantation. Subsequently, rheological studies were performed on hydrogels. Storage modulus (G′) and loss modulus (G″) respectively reflected the change of viscosity and elasticity, and both of them increased with temperature (Additional file 1:Figure S1A and Fig. 2C). Below a specific temperature, G′′ was considerably larger than G′, indicating the liquid-state behavior in these cases. By contrast, G′ > G′′ was detected upon further increasing temperature, which is a typical characteristic of the solid-like behavior (Additional file 1: Fig. S1A and Fig. 2C). Notably, G′ > G″ at 37 °C, suggesting a gel formation (Figure S1A and Fig. 2C). Moreover, frequency sweep at 37 °C confirms higher G′ compared to G″ for hydrogels, which showed that the formulation exhibited viscoelastic behavior (Fig. 2D, Additional file 1: Fig. S1C). Viscosity measurements were also performed, and the results showed that hydrogels exhibited a typical shear-thinning behavior (non-Newtonian) and the viscosity decreased as a function of shear rate (Fig. 2E, Additional file 1: Fig. S1E).

PF127 hydrogels have previously been reported to slow the release of incorporated drugs [38], which supports our finding that cumulative release of 80% of the ropivacaine took more than 12 h (Fig. 2F). However, high concentrations of ropivacaine in the circulation can cause convulsions and even death in mice [8]. Hence, we tested the biosafety of PFR in mice after tumor resection (Fig. 2G). As shown in Fig. 2H, injection of free ropivacaine at the wound site resulted in convulsions in 16.67% of mice, while convulsions did not occur in the PFR-administered mice. This suggests that PFR has good biosafety and may be used for the prolonged relief of POP.

Evaluation of engineered LAs for analgesia in mice after tumor resection is still pending. Therefore, we tested the analgesic effects of the PFR in postoperative mice. After tumor resection, mice were administered free ropivacaine, or PFR for postoperative analgesia. Thermal hyperalgesia in mice is commonly used to simulate POP in rodents [39], and in our assessment, nociception in the hind paws of the model mouse in response to a thermal stimulus was tested using a removable thermal stimulator [30] (Fig. 2J). The results show that in comparison to the control and free ropivacaine analgesia, PFR analgesia significantly increased PWL for more than 16 h (Fig. 2K). Notably, PF127 hydrogels itself had no analgesic effect (Additional file 1: Fig. S2). Furthermore, it is well known that activated microglia in the dorsal horn of the spinal cord contribute to POP, and phosphorylated p38 mitogen-activated protein kinase (MAPK, p-p38) is a marker of activated microglia [40]. We observed many p-p38 positive cells in dorsal horn of control and ropivacaine-treated mice after surgery, but there were less p-p38 positive cells in the PFR-treated mice (Fig. 2L). The statistical results show that compared to control and ropivacaine analgesia, PFR analgesia significantly reduced the number of p-p38 positive cells in the spinal dorsal horn, suggesting reduced activation of microglia (Fig. 2M). Overall, these results demonstrate that PFR provides effective prolonged postoperative analgesia.

### Ropivacaine-loaded hydrogel increases MHC-I levels in vitro

**Fig. 3:**
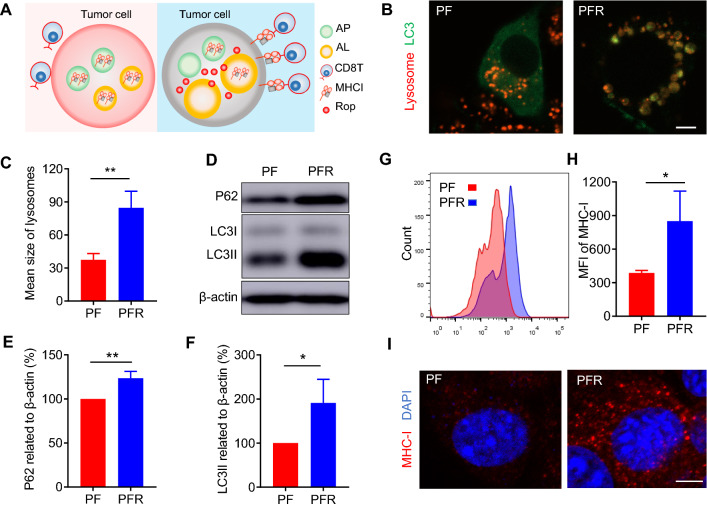
PFR upregulates MHC-I in 4T1 cells. **A** Schematic diagram of MHC-I upregulation by ropivacaine. AP: autophagosome, AL: autolysosome, Rop: ropivacaine. **B** Fluorescent images of LC3 and lysosomes. Ad-GFP-LC3B-infected cells were treated with PF or PFR for 8 h and stained with Lyso-Tracker red (scale bar represents 5 μm). **C** Statistical results of the size of lysosomes from (**B**) (n = 4). **D**–**F** Western blot and statistical results of P62 and LC3II in 4T1 cells treated with PF or PFR for 8 h. **G** Flow cytometry results of the mean fluorescence intensity (MFI) of MHC-I on the 4T1 cell surface. **H** Statistical results from (**G**). **I** Immunofluorescent staining of MHC-I (red) and DAPI (blue) (scale bar represents 5 μm). *PF* PF127 hydrogel, *PFR* PF hydrogel mixed with ropivacaine

Postoperative recurrence is a significant challenge for cancer treatment. Although some studies have shown that LAs can directly kill tumor cells [22, 23], studies using LAs for immunotherapy to prevent tumor recurrence have not yet been reported. Impaired antigen presentation caused by the loss of MHC-I is a common mechanism of immune evasion by tumor cells [24]. Keisuke et al. found that MHC-I was selectively targeted for lysosomal degradation via an autophagy-dependent mechanism; however, inhibition of autophagy restores MHC-I levels and leads to improved antigen presentation, enhanced anti-tumor T cell responses, and reduced tumor growth in mice [25]. Interestingly, our previous study demonstrated that ropivacaine impaired autophagic lysosomal degradation of tumor cells [26], suggesting that ropivacaine-doped hydrogel may upregulate MHC-I levels in tumor cells (Fig. 3A). To verify this, we tested the effect of PFR on autophagy in 4T1 cells. As shown in Fig. 3B, PFR treatment induced many enlarged autolysosomes, labeled by co-staining with LC3 and lysosomes. Statistical analysis showed that PFR treatment significantly increased the size of lysosomes (Fig. 3C), which suggests a dysfunction of autophagy [41] (Fig. 3B, C). LC3II is a marker of autophagosomes [42], while P62 is a protein substrate that is selectively degraded by autophagy [43]. We found that PFR treatment significantly increased the levels of P62 and LC3II (Fig. 3D–F), revealing normal initiation of autophagy but impairment of autophagic lysosomal degradation. Next, we tested MHC-I levels in 4T1 cells. The flow cytometry results showed that PFR treatment significantly increased the mean fluorescence intensity (MFI) of MHC-I (Fig. 3G, H), and the confocal images also showed a stronger MHC-I signal in PFR-treated cells compared with those treated with PF (Fig. 3I). These results demonstrated that PFR treatment significantly increased MHC-I levels in 4T1 cells.

### Ropivacaine-loaded hydrogel prevents tumor recurrence

**Fig. 4:**
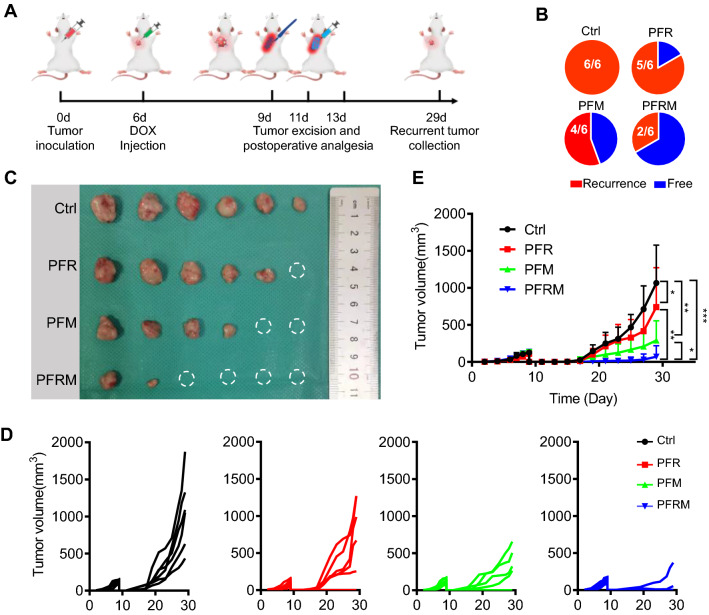
PFRM analgesia inhibits postoperative tumor recurrence. **A** Schematic diagram showing the experimental surgical procedure. **B** Proportion of tumor recurrence under different postoperative analgesia. **C** Image of exfoliated tumors. **D** Individual and **E** average tumor growth curves under different postoperative analgesia (n = 6). *PFR* PF hydrogel mixed with ropivacaine, *PFM* PF hydrogel mixed with imiquimod, *PFRM* PF hydrogel mixed with ropivacaine and imiquimod

**Fig. 5:**
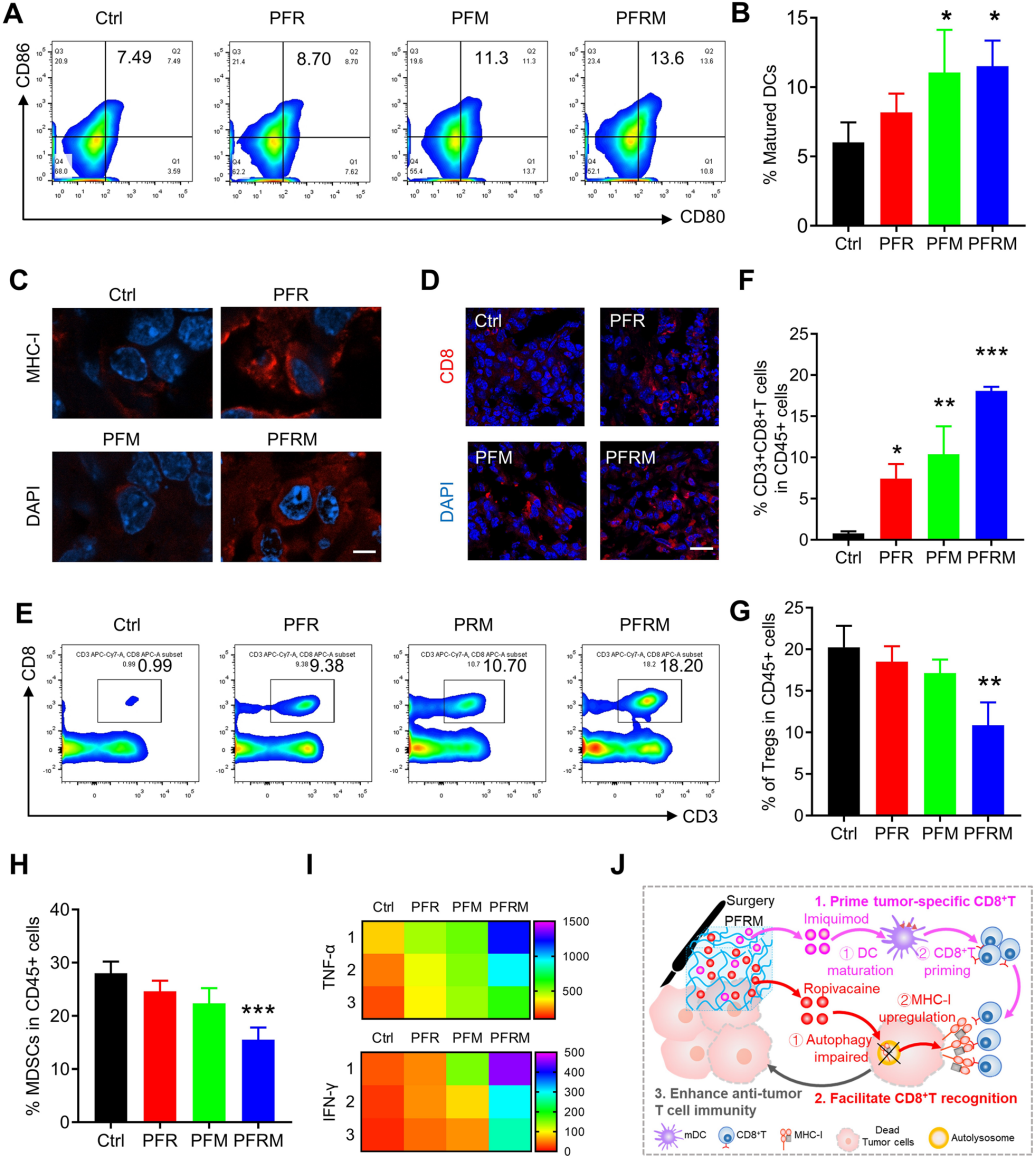
PFRM increases CD8^+^T cell infiltration. **A**–**B** Flow cytometry analysis of the percentage of CD80^+^ and CD86^+^ DCs in draining lymph nodes (n = 3). **C** Immunofluorescence staining of MHC-I in recurrent tumors (scale bar represents 5 μm). **D** Immunofluorescence staining of CD8^+^ T cells in recurrent tumor (scale bar represents 20 μm). **E**, **F** Flow cytometry analysis of CD3^+^ and CD8^+^ T cells in recurrent tumor gating of CD45^+^ cells (n = 3). **G**, **H** Flow cytometry analysis of Tregs cells and MDSCs in recurrent tumor gating of CD45^+^ cells (n = 3). **I** Levels of TNF-α and IFN-γ in tumors (n = 3). **J** Schematic diagram of PFRM analgesia triggering an immune response. Imiquimod primes the DCs maturation, resulting in an increased population of CD8^+^ T cells in residual tumor tissues. Ropivacaine upregulates MHC-I in residual tumor cells, facilitating the recognition of CD8^+^ T cells to tumor cells. *PFR* PF hydrogel mixed with ropivacaine, *PFM* PF hydrogel mixed with imiquimod, *PFRM* PF hydrogel mixed with ropivacaine and imiquimod

The upregulation of MHC-I can promote the recognition of tumor cells by cytotoxic T lymphocytes (CD8^+^ T cells) [25], while CD8^+^ T cells can be primed by imiquimod through promoting DC maturation [44]. Therefore, ropivacaine and imiquimod co-doped PF hydrogel (PFRM) may maximize the CD8^+^ T cells infiltrations into residual tumor cells after surgery. Hence, we assessed the effect of PFRM analgesia on immunotherapy for postoperative recurrence using a mouse model that was sequentially injected with chemotherapeutic drugs to control tumor growth and resected the tumor under general anesthesia. We found that additional introduction of imiquimod did not change the rheological properties of PF127 hydrogels (Additional file 1: Fig. S1). Besides, the drug-loaded hydrogels could be retained in vivo for about 48 h (Additional file 1: Figs. S3, S4). Then, PFR, PFM or PFRM was administered for three times to manage POP (Fig. 4A). Twenty days after tumor resection, we found that all tumors recurred in the control mice. However, the tumor recurrence rates were 5/6, 4/6, and 2/6 in mice treated with PFR, PRM, and PFRM, respectively. These results suggest an excellent tumor-recurrence inhibitory effect of postoperative analgesia using PFRM (Fig. 4B). Furthermore, continuous tumor measurement showed mild inhibition of tumor growth by PFR or PFM analgesia, while PFRM analgesia resulted in a strong anti-tumor effect (Fig. 5C–E). Moreover, PFRM analgesia showed a significant antitumor effect compared to PFR or PFM analgesia (Fig. 5C–E). Notably, PF127 hydrogels itself had no antitumor effect (Additional file 1: Fig. S5). In addition, there were no significant differences in mouse body weight and physiological structures of the main organs after different modes of analgesic management (Additional file 1: Figs. S6 and S7), indicating good biosafety of PFRM.

### Mechanism validation of inhibition of tumor recurrence

The mechanism of anti-tumor recurrence by PFRM analgesia was further investigated. As shown in Fig. 5A, B, the percentage of CD80^+^ and CD86^+^ cells was significantly increased in the draining lymph nodes of PFM- and PFRM-treated mice compared to those of control mice, revealing increased DC maturation. Furthermore, MHC-I was upregulated in PFR- and PFRM-treated mouse tumor cells (Fig. 5C). Consequently, PFR- and PFM-treated mice showed mild CD8^+^ T cell infiltration, while PFRM analgesia resulted in maximum CD8^+^ T cell infiltration (Fig. 5D–F). In addition, PFRM analgesia significantly reduced the inhibitory immune cells populations in tumors, including Tregs and MDSCs (Figure S8 and Fig. 5G, H). Moreover, PFRM analgesia also remarkably increased the levels of TNF-α and IFN-γ compared to control mice (Fig. 5I). Collectively, PFRM analgesia promoted the infiltration of CD8^+^ T cell and elicited a strong immune response in residual tumor tissues, resulting in the inhibition of tumor recurrence (Fig. 5J).

## Conclusions

In summary, postoperative analgesia with ropivacaine-loaded Pluronic F127 hydrogel reduce the incidence of high-dose ropivacaine-induced convulsions and prolongs postoperative pain relief for more than 16 h. In addition, postoperative analgesia with ropivacaine-loaded hydrogel upregulates MHC-I in tumor cells by impairing autophagy, consequently promoting CD8^+^ T cell infiltration and preventing tumor recurrence. Overall, this study for the first time provides an LA-based approach for simultaneous long-lasting postoperative analgesia and prevention of tumor recurrence.

## Supplementary Information

**Additional file 1: Figure S1.** Rheological properties of hydrogels. Temperature-dependent rheology of (A) PF and (B) PFRM aqueous dispersion. Frequency-dependent rheology of (C) PF and (D) PFRM hydrogel at 37°C. The shear-thinning behavior of (E) PF and (F) PFRM hydrogel indicated by steady-shear rheology. **Figure S2.** Paw withdrawal latency was measured at 0, 3, 10, 16, and 24 h after tumor resection with PBS or PF127 hydrogel treatment (n=3). **Figure S3.** (A) Ropivacaine concentrations in tumor tissues were measured at indicated time pointes. (B) Fluorescence images of mice at the indicated times after subcutaneous injection of ICG loaded PF127 hydrogel. **Figure S4.** (A) Degradation kinetics of PFRM hydrogel formulations incubated at 37 °C measured by weight remaining (%). (B) Degradation of PFRM hydrogel* in vivo*. The PFRM hydrogel was injected into mice subcutaneously and photos around the hydrogels were taken at 5 minutes, 12 hours and 48 hours after injection. **Figure S5.** (A) Image of exfoliated tumors after PBS or PF treatments. (B) Average tumor growth curves under PBS or PF treatment (n=3). PF: PF127 hydrogel. **Figure S6.** Body weight changes of mice under different treatments. **Figure S7.** HE staining of main organs after different treatments. **Figure S8.** (A) Flow cytometry analysis of Tregs. (B) Flow cytometry analysis of MDSCs.

## Data Availability

All data generated and analyzed during this research are included in this published article and its additional file.
