# Supplementary material for: Long-lasting postoperative analgesia with local anesthetic-loaded hydrogels prevent tumor recurrence via enhancing CD8+T cell infiltration
Source: J Nanobiotechnology. 2023 Feb 10;21:50. doi: 10.1186/s12951-023-01803-8 (PMC9912655; doi:10.1186/s12951-023-01803-8)
Supplement: Supplementary file 1 — Additional file 1: Figure S1. Rheological properties of hydrogels. Temperature-dependent rheology of (A) PF and (B) PFRM aqueous dispersion. Frequency-dependent rheology of (C) PF and (D) PFRM hydrogel at 37°C. The shear-thinning behavior of (E) PF and (F) PFRM hydrogel indicated by steady-shear rheology. Figure S2. Paw withdrawal latency was measured at 0, 3, 10, 16, and 24 h after tumor resection with PBS or PF127 hydrogel treatment (n=3). Figure S3. (A) Ropivacaine concentrations in tumor tissues were measured at indicated time pointes. (B) Fluorescence images of mice at the indicated times after subcutaneous injection of ICG loaded PF127 hydrogel. Figure S4. (A) Degradation kinetics of PFRM hydrogel formulations incubated at 37 °C measured by weight remaining (%). (B) Degradation of PFRM hydrogel in vivo. The PFRM hydrogel was injected into mice subcutaneously and photos around the hydrogels were taken at 5 minutes, 12 hours and 48 hours after injection. Figure S5. (A) Image of exfoliated tumors after PBS or PF treatments. (B) Average tumor growth curves under PBS or PF treatment (n=3). PF: PF127 hydrogel. Figure S6. Body weight changes of mice under different treatments. Figure S7. HE staining of main organs after different treatments. Figure S8. (A) Flow cytometry analysis of Tregs. (B) Flow cytometry analysis of MDSCs. [file 12951_2023_1803_MOESM1_ESM.docx]

**Additional file**

**Materials and Methods**

**1. *In vitro* hydrogel degradation**

The *in vitro* degradation of hydrogels was determined by performing a weight remaining (%) experiment, as described by previous literature [1]. Briefly, samples of 500 μL of PFRM hydrogel formulations were added into glass vials and placed at 37 °C. After gelation, the original weight of each hydrogel formulation was recorded as W0. 1 mL of pre-equilibrated PBS (pH 7.4) was gently laid over the surface of the hydrogels and incubated in a metal bath at 37 °C. The weight of the remaining hydrogel samples (Wt) was recorded at regular time intervals (1, 3, 6, 9, 12, 24, 36, and 48 h) after removing the supernatant. The *in vitro* degradation assay was performed in triplicate. The weight remaining (%) was calculated as:

𝑊𝑒𝑖𝑔ℎ𝑡 𝑟𝑒𝑚𝑎𝑖𝑛𝑖𝑛𝑔 (%) = 𝑊𝑡 / 𝑊0 × 100%

**2. Degradation of hydrogel *in vivo***

150 μL of PFRM hydrogel subcutaneously injected into the right breast pad of BALB/c mice. At indicated timepoints, the mice were sacrificed, and the residual hydrogel in the subcutaneous layer was photographed.

**3. Near-infrared fluorescence imaging**

ICG-loaded PF127 hydrogel was subcutaneously injected. Mice were anesthetized by isoflurane, and then fluorescent images of mice were collected using an IVIS® Spectrum system (Caliper, Hopkington, MA, USA) at the indicated time points.

**4. Determination of ropivacaine contents in vivo**

4T1 cells (8 × 10^5^, 100 μL) were inoculated into the right breast pad of the BALB/c mice and 50 μL of PFR was intratumorally injected when the tumor volume reached approximately 150 mm^3^. At 2, 6, 12 and 48 hours after the injection, mice were killed and the tumor were collected. The tissues were added to three volumes PBS per tissue weight, homogenized, and stored at -20℃ until being analyzed. HPLC analysis was performed on a Shimadzu HPLC system, equipped with LC-20AP binary pump, SPD-20A UV-VIS detector, and Symmetry C18 column.

**Figure S1**

Figure S1. Rheological properties of hydrogels. Temperature-dependent rheology of (A) PF and (B) PFRM aqueous dispersion. Frequency-dependent rheology of (C) PF and (D) PFRM hydrogel at 37°C. The shear-thinning behavior of (E) PF and (F) PFRM hydrogel indicated by steady-shear rheology.

\

**Figure S2**

Figure S2. Paw withdrawal latency was measured at 0, 3, 10, 16, and 24 h after tumor resection with PBS or PF127 hydrogel treatment (n=3).

**Figure S3**

Figure S3. (A) Ropivacaine concentrations in tumor tissues were measured at indicated time pointes. (B) Fluorescence images of mice at the indicated times after subcutaneous injection of ICG loaded PF127 hydrogel.

**Figure S4**

Figure S4. (A) Degradation kinetics of PFRM hydrogel formulations incubated at 37 °C measured by weight remaining (%). (B) Degradation of PFRM hydrogel *in vivo*. The PFRM hydrogel was injected into mice subcutaneously and photos around the hydrogels were taken at 5 minutes, 12 hours and 48 hours after injection.

**Figure S5**

Figure S5. (A) Image of exfoliated tumors after PBS or PF treatments. (B) Average tumor growth curves under PBS or PF treatment (n=3). PF: PF127 hydrogel.

**Figure S6**

Figure S6. Body weight changes of mice under different treatments.

**Figure S7**


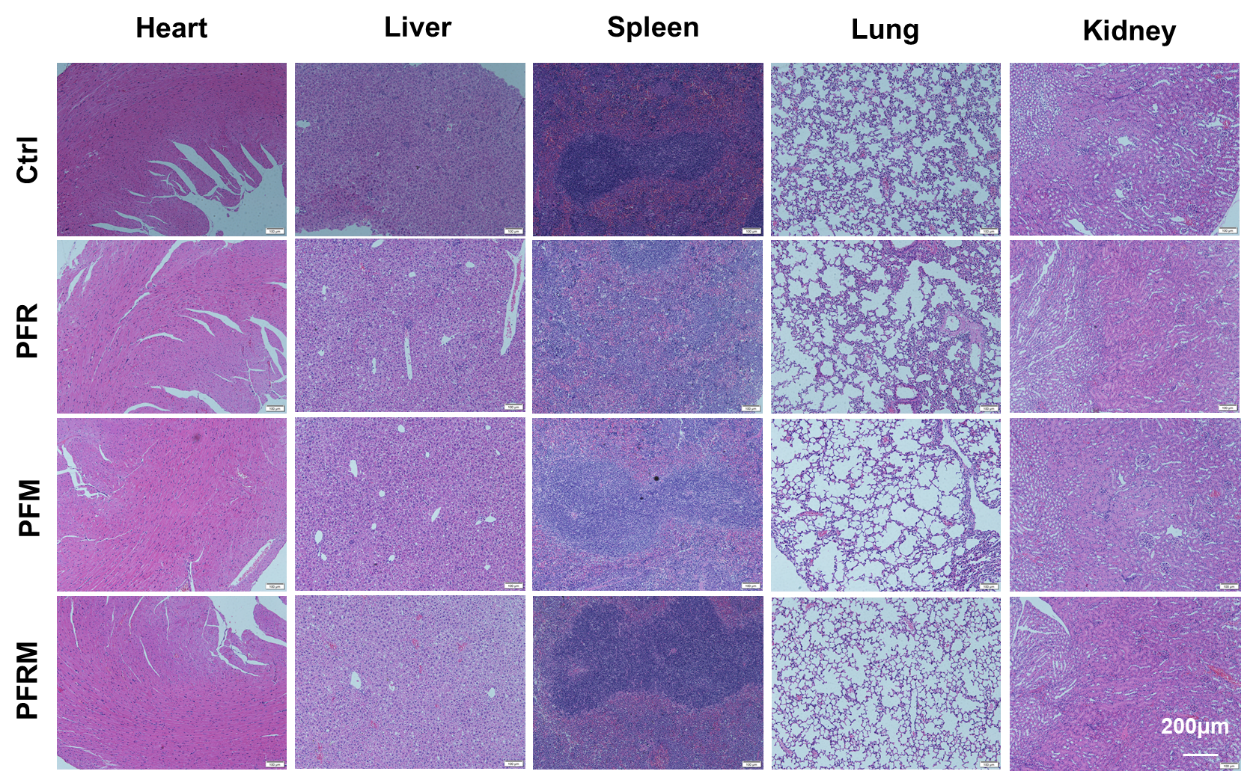


Figure S7. HE staining of main organs after different treatments.

**Figure S8**

Figure S8. (A) Flow cytometry analysis of Tregs. (B) Flow cytometry analysis of MDSCs.

**References**

1. Luo G, Sun Y, Zhang J, Xu Z, Lu W, Wang H, Zhang Y, Li H, Mao Z, Ye S, et al: Nanodefensin-encased hydrogel with dual bactericidal and pro-regenerative functions for advanced wound therapy. Theranostics 2021, 11:3642-3660.
